# Reformvorhaben „Gelegenheits-Onkochirurgie“: Medizinstrategische Analyse und Auswirkungen der Gesetzesinitiative im Rahmen des Krankenhausversorgungsverbesserungsgesetzes (KHVVG) aus der Perspektive eines Universitätsklinikums

**DOI:** 10.1007/s00104-024-02146-2

**Published:** 2024-08-14

**Authors:** Valesca Spreider, Stefan Fichtner-Feigl, Frederik Wenz, Dalibor Bockelmann

**Affiliations:** 1 Stabsstelle Medizinische Strategie und Vernetzung, Universitätsklinikum Freiburg, Breisacher Str. 153, 79110 Freiburg, Deutschland; 2 Klinik für Allgemein- und Viszeralchirurgie, Universitätsklinikum Freiburg, Hugstetter Str. 55, 79106 Freiburg, Deutschland; 3 Universitätsklinikum Freiburg, Breisacher Str. 153, 79110 Freiburg, Deutschland

**Keywords:** Krankenhausreform, Leistungskonzentration, Spezialisierung, Zentralisierung, Onkologie, Hospital reform, Concentration of in-patient treatment, Specialization, Centralization, Oncology

## Abstract

**Hintergrund:**

Nach wie vor stellen Krebsleiden die zweithäufigste Todesursache in Deutschland dar. Leistungssteuerung und Spezialisierungskonzepte in der Medizin bergen Potenziale, die Versorgung und die Überlebenschancen von Patientinnen und Patienten positiv zu beeinflussen.

**Ziel der Arbeit:**

Aus der Gesetzesinitiative im Rahmen des KHVVG resultiert aus der Perspektive des UKF eine ganze Reihe medizinstrategischer Implikationen. Diese Arbeit erläutert und diskutiert die Hintergründe, Ziele und Inhalte des Reformvorhabens „Gelegenheits-Onkochirurgie“ und gibt Perspektiven auf strategische Handlungsfelder.

**Material und Methoden:**

Analyse und Interpretation des Entwurfs eines Gesetzes zur Verbesserung der Versorgungsqualität im Krankenhaus und zur Reform der Vergütungsstrukturen (Gesetzentwurf der Bundesregierung).

**Ergebnisse:**

Aus Sicht des UKF sollten Krankenhäuser frühestmöglich und proaktiv Kooperationsgespräche mit benachbarten Krankenhäusern zur Gestaltung der regionalen Gesundheitsversorgung führen mit den Zielen, die lokale Allokation onkologischer Patientinnen und Patienten im Sinne einer bestmöglichen Behandlung abzubilden, den Verlust von Patienten an den betroffenen Standorten abzufedern sowie den Patientenaufwuchs bei den Einrichtungen, die weiterhin onkochirurgisch versorgen werden, vorzubereiten.

**Diskussion:**

Das noch laufende Gesetzgebungsverfahren sowie der Umstand, dass eine belastbare Analyse der relevanten Leistungsbereiche erst im ersten Halbjahr 2025 für Krankenhäuser möglich sein wird, stellt die Einrichtungen vor besondere Herausforderungen in der strategischen Planung ihrer Aktivitäten. Die im Rahmen dieser Arbeit dargestellten Lücken im Gesetzesvorhaben sollten dringend geschlossen werden, um die Ziele des Vorhabens nicht zu konterkarieren und die im System verbleibenden Leistungserbringer bei ihren Vorbereitungen zu unterstützen.

## Abstract (en)

**Background:**

Cancer remains the second most common cause of death in Germany. Performance management and specialization concepts in medicine have the potential to positively influence the care and chances of survival of patients.

**Objective:**

From the perspective of the University Hospital Freiburg (UKF), the legislative initiative within the framework of the Hospital Treatment Improvement Act (KHVVG) results in a number of medical strategic implications. This article explains and discusses the background, objectives and contents of the reform project “Occasional surgical oncology” and provides perspectives on strategic fields of action.

**Material and methods:**

Analysis and interpretation of the draft of the Act for improvement of the treatment quality in hospitals and on the reform of the remuneration structures (Federal Government draft act).

**Results:**

From the point of view of the UKF hospitals should engage in cooperative discussions with neighboring hospitals at the earliest opportunity to shape regional healthcare with the goals of mapping the local allocation of oncology patients for optimal treatment, mitigating the loss of patients at affected locations and preparing for patient growth at facilities that will continue to provide treatment in surgical oncology.

**Discussion:**

The ongoing legislative process and the fact that a reliable analysis of relevant treatment areas will be possible for hospitals in the first half of 2025, presents particular challenges for hospitals and the strategic planning of activities. The gaps in the bill presented in this article should be urgently addressed to avoid undermining the project’s goals and to support the hospitals remaining in the healthcare system in their preparations.

## Hintergrund

Nach wie vor wird Krebs als zweithäufigste Todesursache in Deutschland in der Todesursachenstatistik des Statistischen Bundesamtes geführt [1]. Die Zahl der Neuerkrankungen steigt zudem stetig an [2]. Dass eine Leistungssteuerung und Spezialisierungskonzepte in der Medizin Potenziale bergen, die die Versorgung und die Überlebenschancen von Patientinnen und Patienten positiv beeinflussen, scheint bereits gesundheitspolitische Aufmerksamkeit zu genießen: So dient beispielsweise der gesetzliche Auftrag an den Gemeinsamen Bundesausschuss (G-BA), elektive stationäre Leistungen zu identifizieren und zu katalogisieren, bei denen ein evidenter Zusammenhang zwischen medizinischer Behandlungshäufigkeit und Ergebnisqualität besteht (Mindestmengen; [3]). Für die Krebsmedizin im Speziellen zeigte nicht zuletzt das durch den Innovationsfonds beim G‑BA geförderte Projekt „WiZen“ (Wirksamkeit der Versorgung in onkologischen Zentren), wie sich „Zentreneffekte“ auf die Überlebenschancen Tumorekrankter auswirken: Basierend auf bundesweiten GKV-Abrechnungsdaten sowie Daten aus vier klinischen Krebsregistern konnte demonstriert werden, dass die Erstbehandlung elf unterschiedlicher Krebserkrankungen in zertifizierten Zentren im Vergleich zu nichtzertifizierten Einrichtungen durchweg mit einer niedrigeren Mortalität assoziiert ist. Die relativen Überlebensvorteile lagen bei den verschiedenen Entitäten zwischen 3 und 23 % [4]. Darüber hinaus konnten positive Effekte innerklinischer Qualitätsindikatoren im Zusammenhang mit der Behandlung in einem nach den Kriterien der Deutschen Krebsgesellschaft zertifizierten Zentrum gezeigt werden, wie bspw. ein verbessertes Komplikationsmanagement und eine sinkende Krankenhaussterblichkeit [5, 6].

**Abb. 1:**
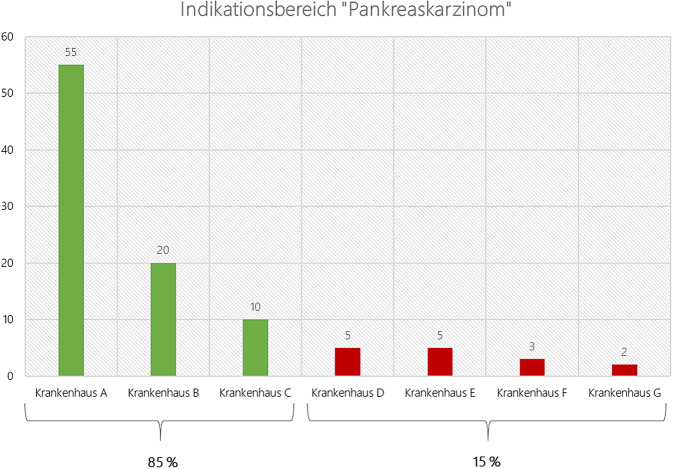
Theoretische Darstellung der 85-/15-Logik

Um die Konzentrationsbestrebungen auf dem Gebiet der onkochirurgischen Versorgung zu fördern, sieht der Gesetzentwurf des KHVVG die Einführung des neuen § 40 Krankenhausfinanzierungsgesetz (KHG) vor. Die Regelung normiert Instruktionen an das Bundesinstitut für Arzneimittel und Medizinprodukte (BfArM) und das Institut für das Entgeltsystem im Krankenhaus (InEK), um die Spezialisierung und Bündelung bei der Erbringung chirurgischer Leistungen im Zusammenhang mit einer onkologischen Diagnose (onkochirurgische Leistungen) zu unterstützen. Ausweislich der Begründung im Gesetzentwurf des KHVVG soll ab dem Jahr 2027 ein wesentlicher Anteil der Finanzierung für onkochirurgische Leistungen in einem onkochirurgischen Indikationsbereich für Krankenhäuser mit geringen Fallzahlen, die zusammen 15 % der Fälle des einschlägigen Indikationsbereichs erbringen, entfallen, wodurch eine Leistungsverlagerung in der Versorgungslandschaft zu erwarten ist. Eine plakative Darstellung dieser Logik ist in der Abb. 1 dargestellt.

Der Gesetzgeber statuiert im Gesetzentwurf die Intension, durch die Bündelung von Ressourcen und Expertise in ausgewählten Gesundheitseinrichtungen die Qualität und Effizienz der onkochirurgischen Versorgung weiter zu verbessern. Die Effekte müssen leistungserbringerseitig antizipiert und in entsprechende medizinstrategische Überlegungen zur regionalen Gesundheitsversorgung einfließen.

## Reformziele

Das Gesetzesvorhaben soll den Anreiz setzen, Gelegenheitsversorgung auf dem Gebiet der onkochirurgischen Versorgung zu vermeiden, um die Qualität dieser Behandlungen durch Zentralisierung zu verbessern. Die Hauptziele des Reformvorhabens lassen sich in diesem Kontext auf drei wesentliche Punkte zusammenfassen:

- Förderung einer sachgerechten Konzentration von Versorgungsstrukturen auf dem Gebiet der Onkochirurgie,
- Sicherstellung einer bedarfsgerechten onkochirurgischen Versorgung,
- Gewährleistung einer qualitativ hochwertigen Patientenversorgung durch gezielte Ressourcenallokation und Prozessoptimierung mit dem Potenzial ökonomischer Positiveffekte.

## Bestandteile der Reform

Das Reformvorhaben umfasst nach derzeitigem Stand drei wesentliche Komponenten, darunter die Definition des einschlägigen Leistungsgerüsts, die Identifikation der betroffenen Standorte sowie das Abrechnungsverbot für bestimmte Entgelte.

### Prozessdarstellung Definition des Leistungsgerüsts

Das BfArM und das InEK sind verantwortlich, onkochirurgische Leistungen mithilfe von ICD-Codes und Operationen- und Prozedurenschlüsseln (OPS) zu definieren und diese onkochirurgischen Indikationsbereichen zuzuordnen. Diese Codes werden entsprechend onkochirurgischer Indikationsbereiche kategorisiert, um die Identifizierung und Erfassung onkochirurgischer Leistungen zu erleichtern.

**Abb. 2:**
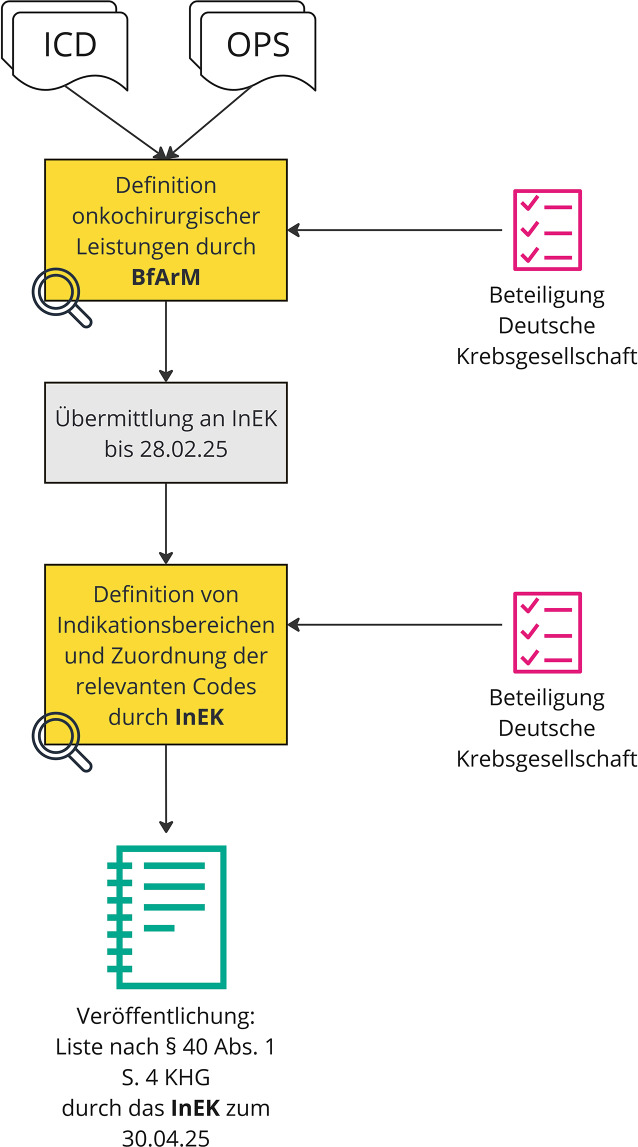
Definition onkochirurgischer Verfahren über ICD- und OPS-Codes

Auf der Basis der Klassifikationsversionen des Jahres 2023 wird das BfArM beauftragt, sämtliche onkochirurgischen Leistungen anhand von ICD- und OPS-Codes zu definieren und diese Aufstellung erstmals zum 28.02.2025 an das InEK zu übermitteln. In einem zweiten Schritt soll sodann das InEK Indikationsbereiche für alle onkochirurgischen Leistungen festlegen. In diesem Kontext ist erwähnenswert, dass eine Stellungnahme bzw. Beteiligung der Deutschen Krebsgesellschaft in beiden Schritten vorgesehen ist, wodurch eine Orientierung an den in der Medizin praxisrelevanten onkologischen Bereichen sichergestellt werden kann. Darüber hinaus erhält das InEK den Auftrag, die ICD- und OPS-Codes aus der Aufstellung des BfArM den Leistungsgruppen und definierten onkochirurgischen Indikationsbereichen zuzuordnen. Eine barrierefreie Veröffentlichung dieses nach Leistungsgruppen und onkochirurgischen Indikationsbereichen differenzierten Verzeichnisses ist erstmalig zum 30.04.2025 auf der Internetseite des InEK zu erwarten. Abb. 2 veranschaulicht den Prozess grafisch.

Ab dem Jahr 2026 soll schließlich eine jährliche Überarbeitung der Datengrundlage (hier: Klassifikationen) und Veröffentlichung der relevanten Liste (im Folgenden: Liste nach § 40 Abs. 1 S. 4 KHG) zum 31.12. eines Jahres durch das InEK erfolgen.

### Prozessdarstellung Standortidentifikation

Das InEK erstellt unter Hinzuziehung der nach Leistungsgruppen und Indikationsbereichen differenzierten Liste nach § 40 Abs. 1 S. 4 KHG (s. Leistungsgerüst) und den Leistungsdaten nach § 21 Krankenhausentgeltgesetz (KHEntgG) eine Auswertung zur Identifikation aller Krankenhausstandorte, die in 2023 onkochirurgische Leistungen erbracht haben.

Kongruent wird auch bei den sog. § 21-er Daten auf das Jahr 2023 Bezug genommen. Sind die Krankenhausstandorte, welche im Jahr 2023 Leistungen der Liste nach § 40 Abs. 1 S. 4 KHG erbracht haben, identifiziert, werden diese je onkochirurgischem Indikationsbereich aufsteigend nach Fallzahl sortiert. Aus dieser Gesamtheit werden diejenigen Krankenhausstandorte, die die wenigsten Fälle mit onkochirurgischen Leistungen und kumuliert 15 % dieser Fälle aller Krankenhausstandorte in einem onkochirurgischen Indikationsbereich aufweisen, bis zum 31.05.2025 durch das InEK barrierefrei auf dessen Internetseite veröffentlicht (im Folgenden: Liste nach § 40 Abs. 2 S. 2 KHG). Nach dem Wortlaut dieser Regelung ist/sind damit auch der/die Krankenhausstandort/e zu veröffentlichen, mit dessen/deren Fällen die 15 %-Perzentile eines relevanten Indikationsbereichs erreicht bzw. überschritten wird/werden.

**Abb. 3:**
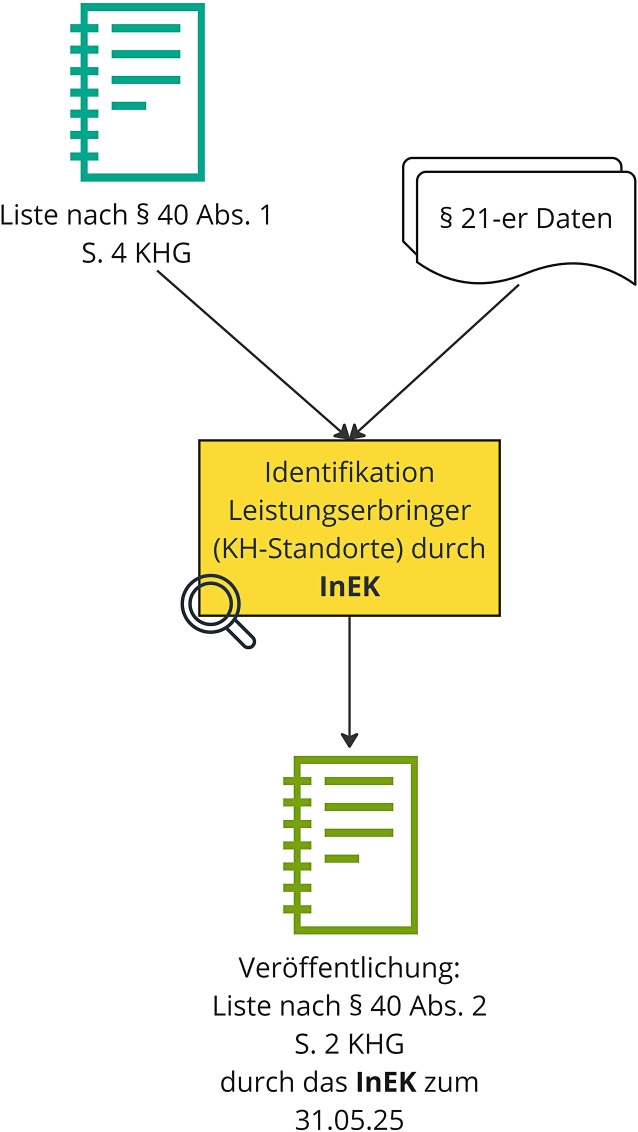
Identifizierung von Krankenhäusern mit geringen onkochirurgischen Fallzahlen

Durch diese Veröffentlichung der Liste nach § 40 Abs. 2 S. 2 KHG erhalten Träger Kenntnis über das für sie geltende Abrechnungsverbot nach § 8 Abs. 4 S. 6 KHEntgG (neue Fassung). Der Prozess der Standortidentifikation wird in Abb. 3 dargestellt.

Die Evaluation eines „Konzentrationseffektes“ soll erstmals zum 30.06.2032 erfolgen. Darüber hinaus sollen o. g. Informationen perspektivisch im Transparenzverzeichnis nach § 135d SGB V verarbeitet werden, um damit Patientinnen und Patienten bei der Suche nach onkochirurgischen Behandlungsangeboten zu unterstützen. Aus Patientensicht werden nur solche Krankenhausstandorte angezeigt, bei denen aufgrund der Häufigkeit der Eingriffe von einer besonderen Expertise der Einrichtung und höheren Behandlungsqualität auszugehen ist.

### Finanzielle Restriktionen (Abrechnungsverbot)

Eine neue Bestimmung in § 8 Abs. 4 des Krankenhausentgeltgesetzes (KHEntgG) soll ein Abrechnungsverbot für bestimmte Leistungen einführen, die in Zusammenhang mit der Erbringung onkochirurgischer Leistungen stehen. Ab dem Jahr 2027 ist vorgesehen, Krankenhausstandorten für Fälle, bei denen Leistungen in einem onkochirurgischen Indikationsbereich der Liste nach § 40 Abs. 1 S. 4 KHG (Leistungsgerüst) erbracht werden und der Krankenhausstandort auf der Liste nach § 40 Abs. 2 S. 2 KHG (Standortidentifikation) benannt ist, die Abrechnungsmöglichkeit der Entgelte nach § 7 Abs. 1 S. 1 Nr. 1–6 sowie Nr. 8 KHEntgG faktisch zu entleeren. Bei Einbezug aller im Gesetzentwurf des KHVVG dargestellten Änderungsvorschlägen sollen nicht mehr vergütet werden:

- Fallpauschalen und damit die zukünftigen rDRG (§ 7 Abs. 1 S. 1 Nr. 1 KHEntgG),
- bundeseinheitlich bepreiste Zusatzentgelte (§ 7 Abs. 1 S. 1 Nr. 2 KHEntgG),
- unbepreiste Zusatzentgelte (§ 7 Abs. 1 S. 1 Nr. 3 KHEntgG),
- Zu- und Abschläge (§ 7 Abs. 1 S. 1 Nr. 4 KHEntgG),
- Entgelte für besondere Einrichtungen (§ 7 Abs. 1 S. 1 Nr. 5 KHEntgG),
- Entgelte für neue Untersuchungs- und Behandlungsmethoden (§ 7 Abs. 1 S. 1 Nr. 6 KHEntgG) sowie
- krankenhausindividuelle Tagesentgelte (neu: § 7 Abs. 1 S. 1 Nr. 8 KHEntgG).

Lediglich die tagesbezogenen Pflegeentgelte zur Abzahlung des Pflegebudgets (§ 7 Abs. 1 S. 1 Nr. 6a KHEntgG), die Vorhaltevergütung zur Abzahlung des Vorhaltebudgets (neu: 7 Abs. § 6b KHEntgG) sowie der Pflegezuschlag (§ 7 Abs. 1 S. 1 Nr. 7 KHEntgG) sollen für Krankenhäuser abrechenbar bleiben. Auf diesem Wege soll die Leistungskonzentration für onkochirurgische Behandlungsangebote und die damit einhergehende Spezialisierung gefördert werden. Gleichzeitig werden etwaige finanzielle Anreize in erheblichem Maße ausgeräumt, was annahmegemäß zu einer Vermeidung onkochirurgischer „Gelegenheitsversorgung“ in der Krankenhauslandschaft führen wird.

## Diskussion

Die Reformierung onkochirurgischer Leistungen im Rahmen des KHVVG stellt grundsätzlich, basierend auf den Erkenntnissen der Mindestmengen- und Zentrumseffekte (s. oben), einen proaktiven Schritt zur Verbesserung der Qualität und Effizienz der onkologischen Versorgung dar. Durch die Förderung von Spezialisierung und Konzentration zielt die Reform darauf ab, den sich wandelnden Bedürfnissen der Patientinnen und Patienten gerecht zu werden und die Ressourcennutzung im Gesundheitssystem zu optimieren. Eine kontinuierliche Einbindung der Stakeholder und Evaluation wird entscheidend sein, um die erfolgreiche Umsetzung und langfristige Wirkung der Reforminitiative sicherzustellen. Allerdings sind unserer Auffassung nach zum jetzigen Zeitpunkt noch eine Reihe an Punkten ungeklärt, die im Kontext des Reformvorhabens jedoch einer Klärung bedürfen: So bleibt beispielsweise der Umgang mit Notfällen bzw. Zufallsbefunden im aktuellen Gesetzentwurf unberücksichtigt und eine Evaluation der Maßnahmen ist erstmals zum 30.06.2032 vorgesehen – vor dem Hintergrund der dynamischen Entwicklungen um die „Krankenhausreform“ ein aus unserer Sicht sehr langer Zeitraum. Weiterhin stellt sich uns die Frage, ob die definierte Größe von 15 % arbiträr festgelegt oder auf einer wissenschaftlichen Herleitung beruht. Sollte ersteres zutreffen, muss diese Zahl aus unserer Sicht nach einer Übergangszeit kritisch auf Effekte evaluiert und bestenfalls auch mit der Deutschen Krebsgesellschaft oder anderen relevanten Fachgesellschaften abgestimmt werden. Unter wissenschaftlichen Gesichtspunkten sollten die Maßnahmen eng im Sinne der tatsächlichen Auswirkungen auf die Behandlungs- und Prozessqualität begleitet, erfasst und perspektivisch näher hinsichtlich der unterstellten Effizienz- und Qualitätssteigerungseffekte in der stationären Versorgung onkologischer Patientinnen und Patienten quantifiziert werden. Dies insbesondere vor dem Hintergrund, dass es sich nach unserer Interpretation um eine einmalige „Bereinigung“ von Versorgungsstrukturen handelt (s. erstmalige Maßnahmenevaluation im Jahr 2032).

Darüber hinaus möchten wir darauf hinweisen, dass zum gegenwärtigen Zeitpunkt keine Regelung zu notwendigen investiven Mitteln in die Infrastruktur solcher Krankenhäuser mitgedacht wird, die perspektivisch mit den Leistungsverlagerungen aus benachbarten Krankenhäusern konfrontiert sein werden. Die Berücksichtigung eines potenziellen „Zentralisierungseffektes“ und folgelogischer monetärer Konsequenzen auf die verbleibenden Leistungserbringer im System findet auch keine explizite Erwähnung in den Fördertatbeständen des neu einzurichtenden Transformationsfonds nach § 12b KHG. Zudem scheint der gesetzgeberische Gedanke, die Leistungskonzentration lediglich an eine mengenmäßige Komponente zu knüpfen, nicht weit genug zu gehen. So könnte darüber nachgedacht werden, die Initiative flankierende Qualitätsanforderungen an die potenziell im System verbleibenden Leistungserbringer zu stellen und gesetzlich zu verankern (z. B. analog Zentrumszertifizierung nach Deutscher Krebsgesellschaft). Auch sollte der Harmonisierungsgrad mit den Zentrumsregelungen des G‑BA näher beleuchtet werden, um etwaige Redundanzen zu identifizieren. Mit dem Regelwerk liegen bereits seit dem Jahr 2020 bundeseinheitliche Kriterien vor, welche als Anforderungen an die Ausweisung und Finanzierung von G‑BA-Zentren formuliert sind (z. B. Mindestfallzahlen, Forschungstätigkeiten, Kooperationen, Art und Anzahl von Fachabteilungen).

Als positiv hervorheben möchten wir die Absicht, die Deutsche Krebsgesellschaft in die Leistungsidentifikation einzubeziehen. Hierdurch ist notwendiger wissenschaftlicher Sachverstand frühzeitig im Prozess repräsentiert. Nach unserer Auffassung könnte die Einflussnahme der Deutschen Krebsgesellschaft durch ihre Stellungnahmen detaillierter im Gesetzestext spezifiziert werden.

### Strategische Handlungsfelder aus Sicht des Universitätsklinikums Freiburg (UKF**)**

- Analyse des Versorgungsbedarfs in der Region: Unerlässlich ist unseres Erachtens eine frühestmögliche, z. B. datenbankgestützte Begutachtung der krankenhauseigenen Versorgungsregion hin auf die Systemrelevanz onkologischer Leistungsangebote, auch unter Einbezug des Angebots benachbarter Krankenhäuser. Hieraus lassen sich erste Erkenntnisse hinsichtlich der Notwendigkeit der Bildung von Leistungsclustern abschätzen. Problematisch sind dabei die Grenzen der zur Verfügung stehenden Datengrundlagen (z. B. Qualitätsberichte) bis zur in Aussicht gestellten Veröffentlichung durch das InEK im Jahr 2025.
- Regionale intersektorale Vernetzung mit stationären Leistungserbringern: Von zentralem medizinstrategischem Interesse ist aus unserer Perspektive die Intensivierung der Vernetzung mit Partnerkrankenhäusern im Sinne abgestimmter patientenorientierter Schwerpunktsetzungen bis hin zum Austausch von Leistungsgruppen in der Versorgungslandschaft. Krankenhäuser sind angehalten, intelligente Wege zu finden, um auf innovative und medizinisch sinnvolle Art Hand in Hand zum Wohle der onkologischen Patientinnen und Patienten zu handeln. Zum Beispiel könnten vermehrt „Chefarzt-Modelle“ (Führung mehrerer Fachabteilungen in Personalunion) etabliert oder regionale Absprachen zu Leistungsaustauschen getroffen werden (z. B. onkochirurgische Eingriffe gesamthaft an ein zertifiziertes Zentrum, dafür die chirurgische Grund- und Regelversorgung an ein kompetentes, qualitätsgesichertes Partnerkrankenhaus verlagern). Aus- und Weiterbildungskonzepte können in diesem Kontext mitgedacht werden.
- Digitale Infrastruktur ausweiten: Der konsequente Ausbau zur Entwicklung eines Gesundheitssystems, das die Chancen der Digitalisierung für eine neue, leistungsfähigere Form der Gesundheitsversorgung zum Wohle von Patientinnen und Patienten nutzt und die Potenziale der Gesundheitswirtschaft hebt, ist aus unserer Sicht ein Schlüsselfaktor für die erfolgreiche Verwirklichung des Reformvorhabens.
- Flankierende Maßnahmen: Teilweise können die vorgeschlagenen Maßnahmen als Eingriff in das ärztliche Standesrecht interpretiert werden. Unser Appell an dieser Stelle ist daher, auf Bundesebene die entsprechenden berufs- und standesrechtlichen Vorkehrungen zu treffen, um die Zielverwirklichung des Reformvorhabens nicht zu konterkarieren. Dies gilt auch für ungenutzte Chancen im Hinblick auf die Zurverfügungstellung notwendiger finanzieller Mittel zur Ertüchtigung einer bedarfsgerechten Krankenhausinfrastruktur. Darüber hinaus halten wir eine durchgehende wissenschaftliche Begleitung sowie Evaluation der geplanten Maßnahmen für den Erfolg des Reformvorhabens und dessen Weiterentwicklung für unentbehrlich.

## Fazit

Medizinstrategisch bleiben vor dem Hintergrund des noch laufenden politischen Prozesses noch mannigfaltige Unsicherheiten. Unserer Meinung nach sollten Krankenhäuser jedoch bereits jetzt proaktiv das Gespräch mit benachbarten Krankenhäusern suchen und Kooperationsgespräche zur Gestaltung der regionalen Gesundheitsversorgung führen, um die lokale Allokation onkologischer Patientinnen und Patienten im Sinne einer bestmöglichen Behandlung an einem Zentrum optimal abzubilden, den Verlust von Patientinnen und Patienten an den betroffenen Standorten abzufedern sowie auf der anderen Seite den Patientenaufwuchs bei den Einrichtungen, die weiterhin onkochirurgisch versorgen werden, vorzubereiten. Die oben skizzierten Handlungsfelder sind von globaler Natur und können daher, unabhängig von der Versorgungsstufe eines Krankenhauses, Impulse für die leistungserbringerseitige Auseinandersetzung mit dem Reformvorhaben „Gelegenheits-Onkochirurgie“ setzen. Mutmaßlich werden aber insbesondere universitäre Standorte und Maximalversorger mit einem zusätzlichen Aufkommen onkologischer Patienten rechnen müssen, welche über die aktuell vorhandenen – personellen sowie infrastrukturellen – Versorgungskapazitäten hinaus behandelt werden müssen.

Einschränkend muss gesagt werden, dass die relevanten Codes frühestens im ersten Quartal, realistisch auch erst im zweiten Quartal 2025 identifiziert und öffentlichkeitswirksam zugänglich werden; entsprechende Berechnungsmodelle, insbesondere unter regionalen Gesichtspunkten, können erst nach Bekanntwerden der einschlägigen Kodierungen erstellt werden. Eine belastbare detailgenaue Analyse ist damit derzeit nicht möglich. Diese muss, wie oben bereits erwähnt, neben medizinisch-prozessualen Gesichtspunkten und der neu zu organisierenden Ressourcenallokation an den betroffenen Standorten auch zwingend die wissenschaftliche Aufarbeitung des Reformvorhabens zu Qualitäts- und Strukturaspekten und somit seines Gelingens berücksichtigen.
